# 3D-printed dual holder system for simultaneous rat PET scanning: design and influence on quantification

**DOI:** 10.1186/s13550-023-01027-9

**Published:** 2023-09-07

**Authors:** Caroline Bouillot, Sébastien Daligault, Radu Bolbos, Nicolas Costes, Luc Zimmer

**Affiliations:** 1 CERMEP-Imagerie du Vivant, 69 boulevard Pinel, 69003 Lyon, France; 2 Lyon Neuroscience Research Center (CRNL), INSERM, Université Claude Bernard, Lyon, France; 3 Hospices Civils de Lyon, Lyon, France

**Keywords:** Small animal imaging, PET/CT, ^18^F-FDG

## Abstract

**Background:**

The low throughout of small animal positron emission tomography (PET) images acquisitions represents a substantial limitation. The aims of this study were (i) to design a low-cost support for simultaneous dynamic PET scanning of two lying rats and (ii) to study its impact on brain image quantification.

**Results:**

Accuracy of concentration measurement was 5.5% for one phantom in the field of view, and 5.7% for two phantoms measured simultaneously. Ratio concentration between phantoms showed an error of 6.7% ± 5.1% for Solo upper position, 6.7% ± 3.7% for Solo lower position, 5.9% ± 4.3% for Duo upper position, and 7.4% ± 6% for Duo lower position 6.7% for separated measures, and 6.6% for simultaneous measures.

In vivo distribution profiles showed no difference between solo and duo uptakes. Region of Interest quantification in the whole brain showed 4.4% variability solo and 3.5% duo. The quantified test–retest bias was 8% in solo and 5% in duo, and the Intraclass Correlation Coefficient was comparable in solo and duo (0.969 vs. 0.966).

**Conclusions:**

Our results showed that simultaneous scans of two rats in INVEON do not affect quantification. The dual support system will allow us to reduce protocol costs and duration.

## Background

Positron emission tomography (PET) is a powerful tool widely used in preclinical research for in vivo imaging physiological processes. By using specific tracers, PET images can depict and measure changes in metabolic activity as well as local pharmacokinetics processes.

As the use of small animal PET models proliferates, and as most experimental designs require multiple groups and conditions, a larger number of scans is required for many protocols. This number of scans is determined a priori by a power analysis considering the expected effect-size of the parameter measured in the image, and the estimated of its typical error. This error is the result of an experimental variability linked to the PET signal, its corrections and reconstruction processes, and to the biological variability of the phenomenon. Experimental variability can be evaluated by simulation or by test–retest reproduction of the measurements [1, 2]. Although reasonable, this estimate, and the power calculation, leads to relatively large sample sizes of a few dozen individuals. Consequently, interest in high-throughput quantitative PET imaging is growing.

Also, PET radiopharmaceuticals of short-lived tracers with moderate to low molecular activity are particularly expensive to produce and generally only allow (at least for Carbon-11 labeling) a single injectable activity at the output of chemical synthesis. The possibility of carrying out several scans simultaneously for the same synthesis would save considerable time and money.

To produce a high throughput of small animal PET acquisitions, several teams have already proved the benefits of scanning multiple animals simultaneously. Based on commercial devices, Habte et al. [3] and Yagi et al. [4] developed a four mice holder showing the potential use of Siemens INVEON PET/CT system for multiple mice simultaneous imaging without a significant degradation of measurement accuracy. In collaboration with a commercial scanner manufacturer, Greenwood et al. [5] designed a similar multiple mice holder for the use in a Mediso nanoScan PET/CT scanner, showing the benefit of simultaneous imaging. With the advent of 3D-printers enabling the design of novel and robust systems, Carter et al. [6] shared a design for a 3D-printed multi-mouse imaging support, compatible with the Siemens INVEON PET/CT scanner.

To our knowledge, Cheng et al*.* [7] are the only study that demonstrated the feasibility of scanning multiple rats simultaneously, by showing a good reproducibility of measurements, whatever the position on the animals. As the dual rat support system of Cheng et al. was designed to be used on an open PET-only system (Siemens microPET P4), the animals were positioned head-to-head, since the imaging system allowed access to both sides of the field of view (FOV). Contrary to the P4, the PET/CT INVEON scanner used in this work has only one side accessible for the tracer injection. Indeed, the opposite side connected to the CT scanner is shielded for radioprotection purposes. For scanning two rats simultaneously, a dual support system would necessarily be designed so that the two rats are positioned in the same direction on bunk beds, allowing access to both animals’ tail veins. As the positioning of each rat implies an offset from the scanner’s FOV center, the influence on the measurement accuracy must be evaluated.

Therefore, the aims of this study were (i) to design a low-cost support for simultaneous dynamic PET scanning of two lying rats for a microPET-CT system and (ii) to study its impact on brain image measurement accuracy.

## Methods

### Design and development of the rat dual support system

**Fig. 1:**
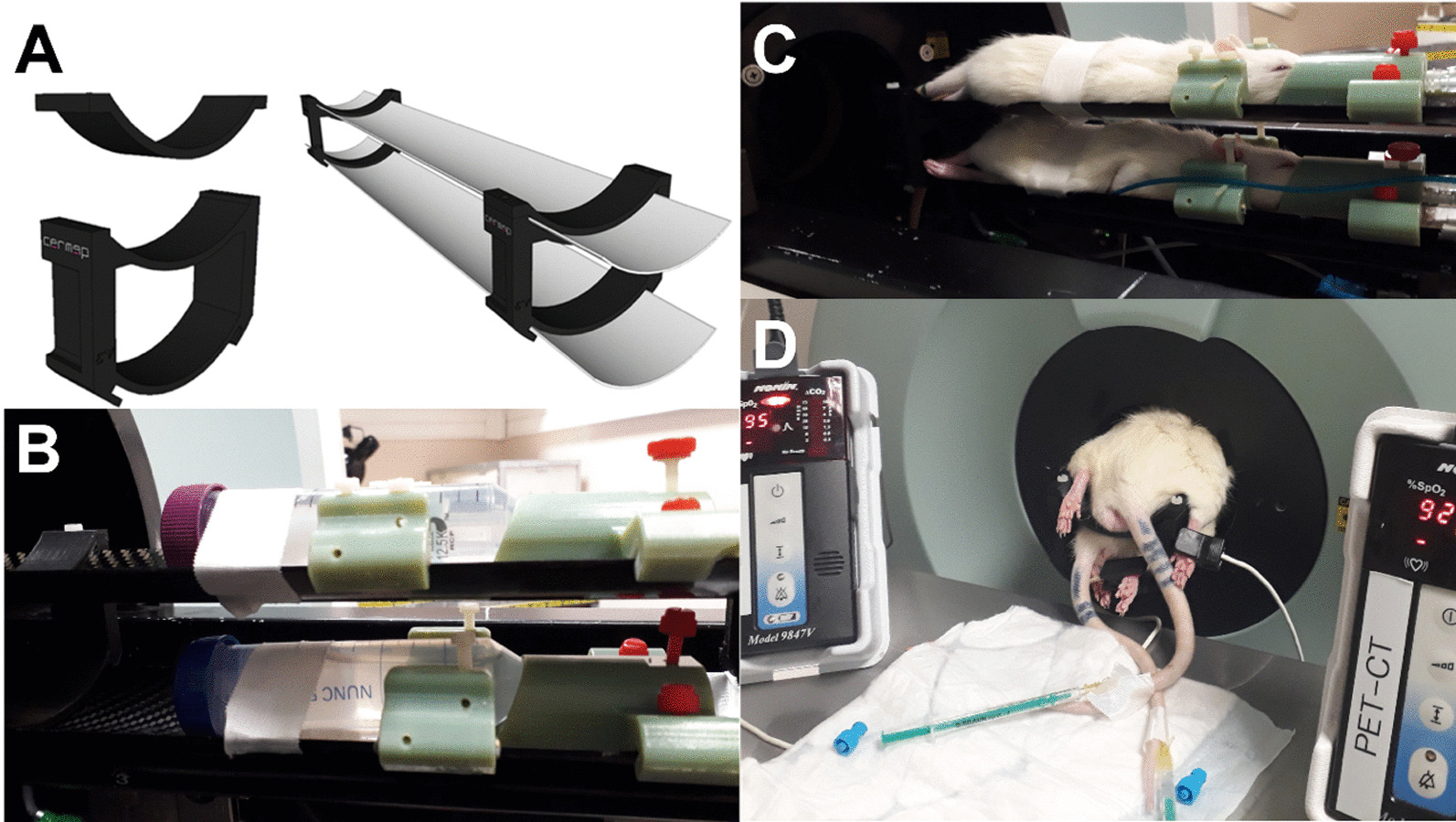
Design of the dual support system. **A** Rendering of 3D-printable support (in black) allowing the superposition of INVEON’s original beds. **B** View of two phantoms within the INVEON dual support bed: in pale green, the designed cone masks and ear bars. **C** View of two installed rats inhaling anesthetic gas. **D** Rats monitoring during PET acquisition

The dual support system was developed for rats and dedicated to Siemens INVEON PET/CT scanner. An open-source computer-aided modeling software (www.blender.org) was employed to generate the 3D files for the design of the dual support system (Fig. 1A), created via a 3D-printer (Raise 3D N2 Plus).

This 3D-printed support system is made of PolyLite™ PLA and composed of two clamping holders allowing to fix and overlap one of the original Siemens INVEON beds 5 cm above the main one, attached to the moving table (Fig. 1B). Both beds are equipped with individual cone masks for anesthesia delivery during PET/CT acquisitions (Fig. 1C). To stabilize the rat’s head and so to avoid any movement, stereotactic ear bars as well as tooth bar were also designed and 3D-printed. In the PET scan position, the rear of the animals remains accessible to the experimenter to administer the radiotracer or any other pharmacological agent, including during the scan (Fig. 1D).

### In vitro PET study

The dual support system was first evaluated in vitro to quantify the accuracy of the radioactivity measurements for various positions in the scanner (upper, lower or dual). For each experiment, two cylindrical phantoms (Falcon tubes) were filled with 50 ml [^18^F]FDG solution with activity differing from about 40%. The mean “low dose” phantom activity was 5.8 ± 0.9 MBq, and the “high dose” phantom activity was 8.5 ± 1.2 MBq.

**Fig. 2:**
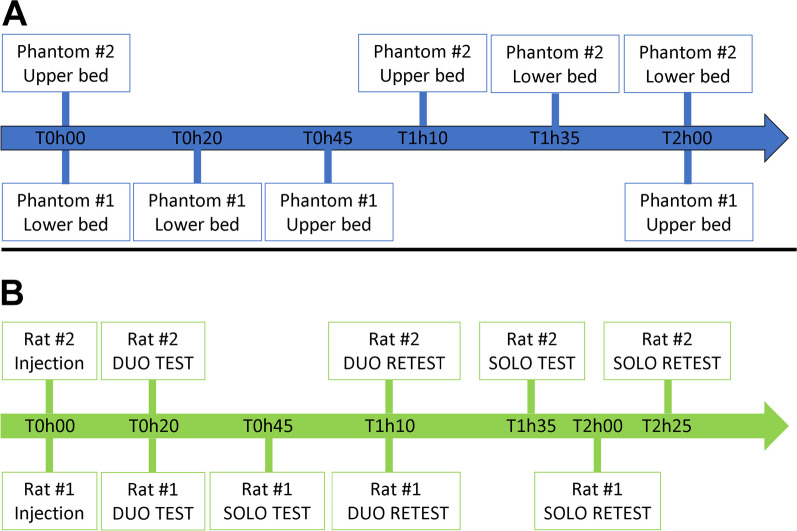
PET experiment timelines: **A** In vitro experiment: a total of 6 sequential imaging acquisitions were performed during the session. **B** In vivo experiment: a total of 6 sequential imaging acquisitions were performed during the session

An example of the timeline of one experiment is shown in Fig. 2A. The scanning conditions were: (1) Duo Test: Phantom #1 (higher activity) in the lower bed and Phantom #2 (lower activity) in the upper bed, (2) Phantom #1 alone, in the lower bed, (3) Phantom #1 alone, in the upper bed, (4) Phantom #2 alone, in the upper bed, (5) Phantom #2 alone, in the lower bed, (6) Phantom #1 in the upper bed and Phantom #2 in the lower bed. Each scan has 10 min duration. The scanning procedure details are given in the in vivo section.

Six experiments were performed. The mean phantom activity ratio was 40% (range 32–52%).

Six images were acquired per experiment, leading to a total of 36 measurements submitted to quantitative analysis.

### In vivo PET study

The dual support system was then evaluated in vivo over four experiments of PET scans with [^18^F]FDG including two rats. All the experiments were conducted in strict accordance with the European Community Council Directive of September 22, 2010 (2010/63/UE) and received approval from the French national ethics committee.

### Animal preparation

Eight Sprague–Dawley adult male rats (Charles River laboratories, France) of 330 ± 66 g were used. Animals were housed in standard temperature and humidity conditions with a 12 h/12 h light/dark cycle. Food and water were provided ad libitum. Four hours prior to the experiments, each couple of rats, were food deprived, but they had free access to water. This restriction was applied to selectively maximize and homogenize the [^18^F]FDG uptake at the brain level, as previously emphasized by Fueger et al. [8]. The animals were anesthetized with 4–5% isoflurane for 5 min (induction phase). A catheter was placed into their caudal vein for radiotracer injection purposes.

The couple of rats were positioned in the dual support system in prone position, the rat #1 underneath the rat #2. During the PET/CT acquisitions, the level of anesthesia was maintained at 2% of isoflurane, with 0.8 L/min air flow rate delivered in a cone mask adapted to rat anatomy (see Fig. 1C). For each rat, the peripheral capillary oxygen saturation level (SpO_2_) was monitored using the Nonin-9847 V veterinary pulse oximetry sensor system (www.nonin.com, Fig. 1D). To constantly maintain the body temperature at a physiological level, both rats were wrapped in a homemade warming sleeve.

### Course of an experiment

After positioning, at T0, the rats received simultaneously a [^18^F]FDG activity of 34.5 ± 5.5 kBq/g, delivered intravenously. Then, each experiment followed the timeline showed in Fig. 2B including six pet scans in the following conditions: (1) Duo Test: both rats scanned simultaneously (2) Rat #1 Solo Test, in the lower bed while rat #2 is removed, (3) Duo Retest: both rats scanned simultaneously, (4) Rat #2 Solo Test, in the upper bed while rat #1 is removed, (5) Rat #1 Solo Retest, in the lower bed, (6) Rat #2 Solo Retest, in the upper bed.

Forty-eight images were analyzed (6 images/experiments, 4 experiments for a total of 8 animals).

### PET/CT imaging procedures

PET imaging was performed using a dedicated small animal PET/CT INVEON system manufactured by Siemens (Erlangen, Germany). The camera has an axial FOV of 12.7 cm and a spatial resolution of 1.8 mm full width at half maximum (FWHM) (in accordance with Bao et al. [7]). Each PET acquisition consisted of a 10 min list mode emission acquisition, followed by a 10 min CT scan using the magnification low acquisition settings for each condition, as represented in Fig. 2. The CT acquisition is used to correct for tissue attenuation and scatter corrections. The acquisition parameters were: attenuation mode; projection: 120; rotation: 200°; binning 4 × 4; effective voxel size: 0.111 × 0.111 × 0.111 mm^3^; voltage: 80 kV; current: 500 µA; filter thickness: 0.5 mm; exposure: 300 ms. PET acquisitions were reconstructed with attenuation and scatter correction by 3D ordinary Poisson ordered subsets expectation maximization (OP-OSEM3D) with 4 iterations and a zoom factor of 1. The reconstructed image is a volume of 159 slices of 128 × 128 matrix voxels, with voxel size 0.4 × 0.4 × 0.8 mm^3^. CT data were reconstructed using a Feldkamp algorithm with a down sample of 2 leading to a reconstructed voxel size of 0.2 × 0.2 × 0.2 mm^3^.

The first PET acquisition (Duo Test) started at T20 timepoint (20 min after [^18^F]FDG injection), followed by the corresponding 10 min CT scan. Then, the Rat #2 was removed from its bed and isolated outside the PET/CT imaging system on a heating pad and maintained under anesthesia. The second PET/CT acquisition of the day was then performed on the Rat #1 alone (Rat #1 Solo Test) at T45 timepoint (45 min after [^18^F]FDG injection). At the end of this acquisition, the Rat #2 was placed back in the imaging system to acquire the third PET/CT scan (Duo Retest), 1h10 post [^18^F]FDG injection. Then, the Rat #1 was removed from its bed and isolated outside the PET/CT imaging system on a heating pad and maintained under anesthesia. The fourth PET/CT scan (Rat #2 Solo Test) was then performed on the Rat #2 alone, 1h35 post [^18^F]FDG injection. Next, the Rat #2 was again removed, and the Rat #1was placed back for the fifth PET/CT acquisition (Rat #1 Solo Retest), 2 h post [^18^F]FDG injection. Lastly, the Rat #1 was removed and returned to its cage. The last acquisition of the afternoon was performed on the Rat #2 alone (Rat# 2 Solo Retest) 2h25 post [^18^F]FDG injection.

### Image post-processing and quantification

Image processing was carried out using the Inveon Research Workplace (IRW 4.2) software (Siemens Medical Solutions USA).

### In vitro study

**Fig. 3:**
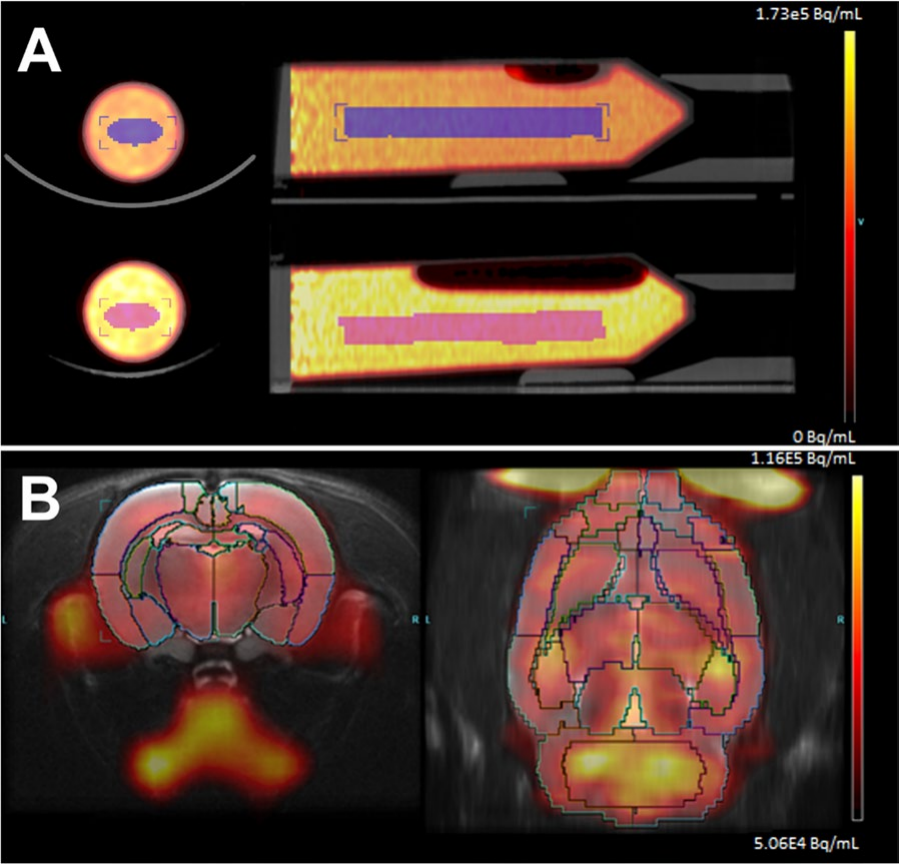
ROI analysis. **A** In vitro experiment showing the cylindrical VOI in the center of the two phantoms. **B** In vivo experiment, zoom on the brain showing the coregistered ROI atlas on the PET images

A cylindrical volume of interest (VOI) of 6 cm^3^ was defined and used to measure the radioactivity concentration in phantoms. With in vitro study, we did not attend to reproduce the NEMA protocol, but rather to use a phantom of size and activity comparable to those used in actual rat studies, and to test the different positions of our device. On these phantoms, an arbitrary VOI of a size that could include a rat brain was arbitrary defined. For all acquisitions, the VOI was placed in the center of the phantom volume, to be equidistant from each edge and to avoid overlapping the air bubbles sometimes residing in the phantom (Fig. 3A). The quantified measurements were expressed in Bq/mL and corrected for decay at the start time of the first imaging acquisition.

*Homogeneity* of the VOI is defined as the coefficient of variation (i.e., standard deviation/mean) of the voxels activities within the VOI, expressed in percent.

### In vivo study

Individual PET images were spatially coregistered over the Lancelot rat brain atlas (Lancelot et al*.* [9]) to allow automatic delineation of 31 brain regions of the atlas (Fig. 3B). The quantified measurements in brain regions were expressed in Standardized Uptake Value:

$${\text{SUV }} = {\text{ Activity }}\;({\text{kBq}}/{\text{mL}})/{\text{Injected Dose }}\;({\text{kBq}})/{\text{rat mass }}\;\left( {\text{g}} \right)$$

where Activity in the mean activity in the region, corrected for decay at the injection time.

Profiles lines of the brain activity were drawn using IRW (Fig. 8B).

Reproducibility of measurements over the various configurations of scanning is first estimated by the linear regression of brain regional activities (31 regions), pooling the subjects [8], taken the *SOLO TEST* or DUO TEST scan as the reference.

In the absence of knowledge of the ground truth of the brain activity, the accuracy of activity measurements was assessed in term of reproducibility indexes between scans—*bias*, *variability* and *the intraclass correlation coefficient (ICC*)—defined as follow:

*Bias* The test–retest bias was calculated as the difference between the test and retest SUV divided by the mean of the test and retest values.

*Variability* is defined as the standard deviation (SD) of the *bias* over subjects. These parameters were expressed as percentage units.

*Reliability.* The measurements' reliability was assessed by *ICC* calculated as the ratio (BSMSS − WSMSS)/(BSMSS + WSMSS) where BSMSS is the mean sum of square between subjects, and *WSMSS* is the mean sum of square within subjects. This statistical ratio estimates the relative contributions of between- and within-subject variability and assumes values from − 1 (i.e., BSMSS = 0) to 1 (identity between test and retest, i.e., WSMSS = 0).

A bias under 10%, a variability of 5% and an ICC over 0.7 are deemed to be acceptable.

Reproducibility indexes were computed at the regional level, from the extracted values of each individual region of the 31 ROIs of Lancelot’s rat brain atlas for all the animals (*n* = 8) across all conditions (Solo test, Solo retest, Duo test, Duo test, Duo retest).

## Results

### In vitro study

**Fig. 4:**
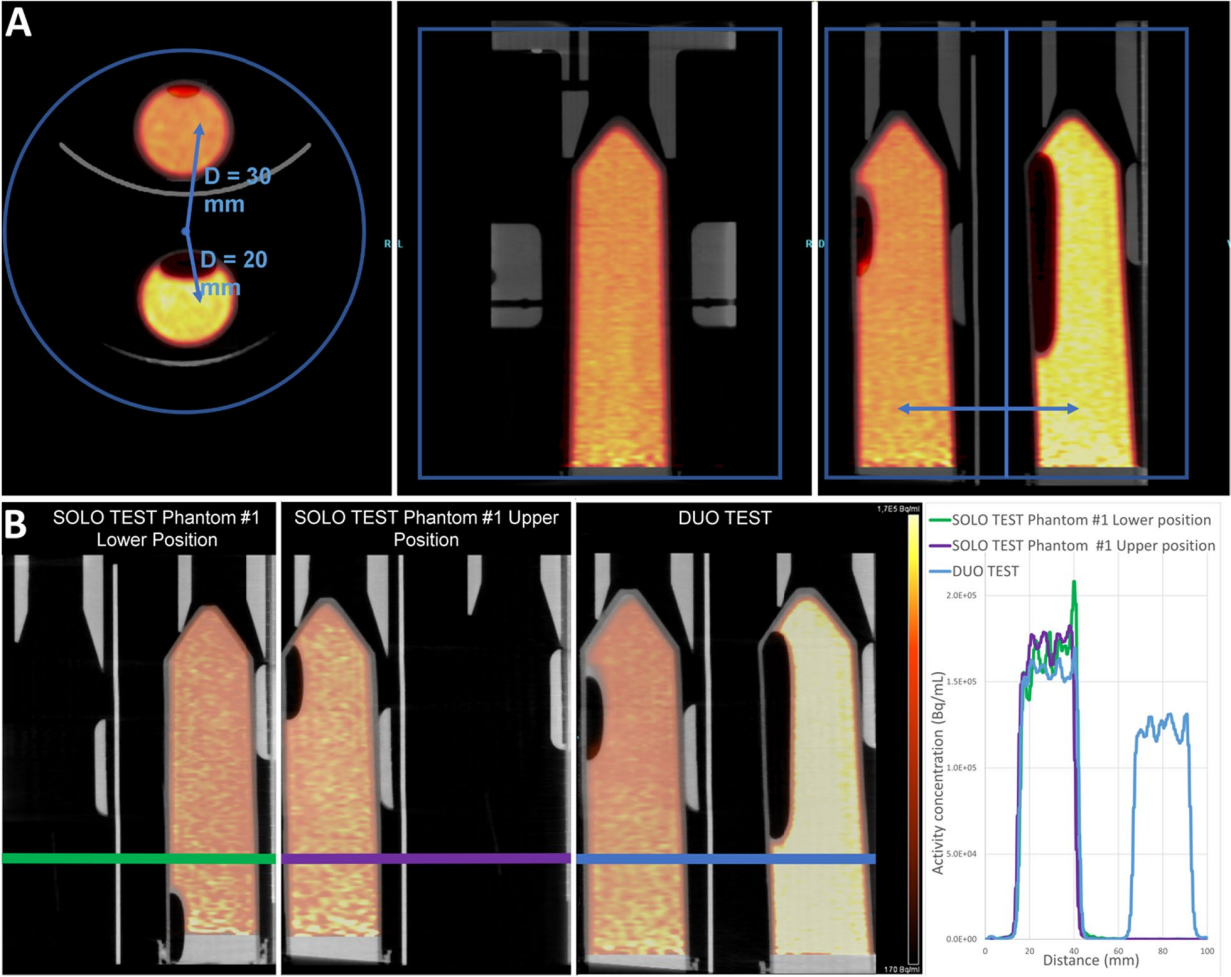
**A** Dual phantoms position within PET FOV. Volumes of interest are located at 20 and 30 mm, respectively, from the center of the FOV. **B** Slices from reconstructed images of phantom #1 scanned in SOLO TEST (Lower Position), SOLO TEST (Upper Position) or DUO TEST. Color-coded profile lines were drawn through the phantoms in all scans (left) to obtain representative plots of radioactivity distribution (right)

The upper phantom has an offset of 30 mm from the center of the FOV, and the lower phantom has an offset of 20 mm (Fig. 4A).

Activity profiles in phantoms are shown in Fig. 4B, with phantoms in lower positions, upper positions, and both lower and upper positions. Visually, the activity profiles have a similar shape.

**Fig. 5:**
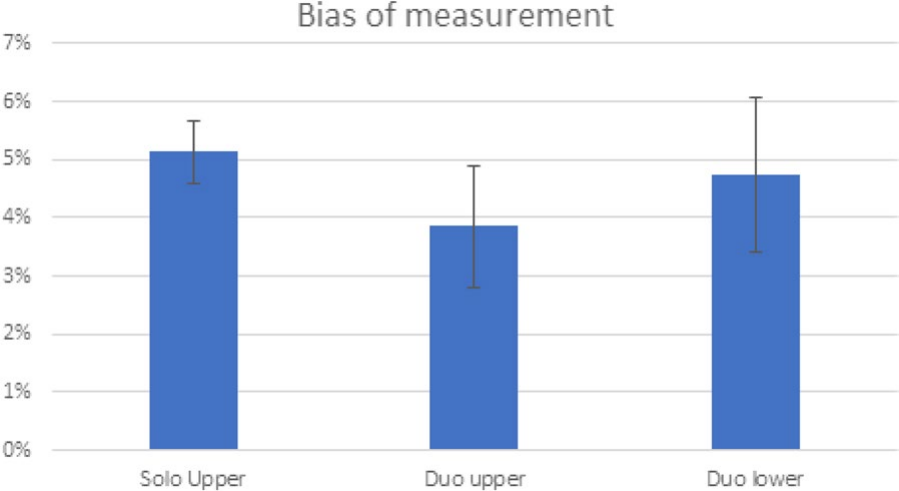
Plot of the bias of the measured activity relative to the activity measured with the phantom in the lower position

Taking the solo lower position as the reference for activity concentration measurements, the bias of the activity measured in the various positions (upper, solo, and duo) was estimated to be under 6% (Fig. 5).

**Fig. 6:**
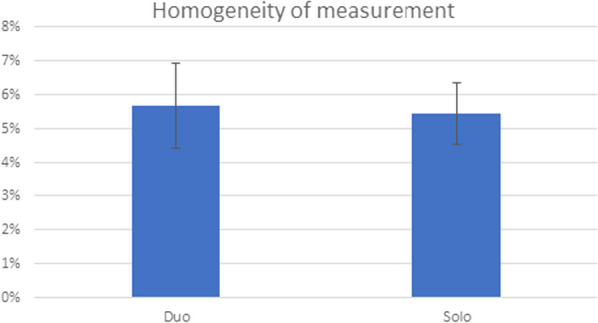
Activity homogeneity in the phantom VOI according to the conditions of measurement

The homogeneity of the VOI was 5.5% ± 0.9% in Solo tests and 5.7% ± 1.3% in Duo tests (Fig. 6).

**Fig. 7:**
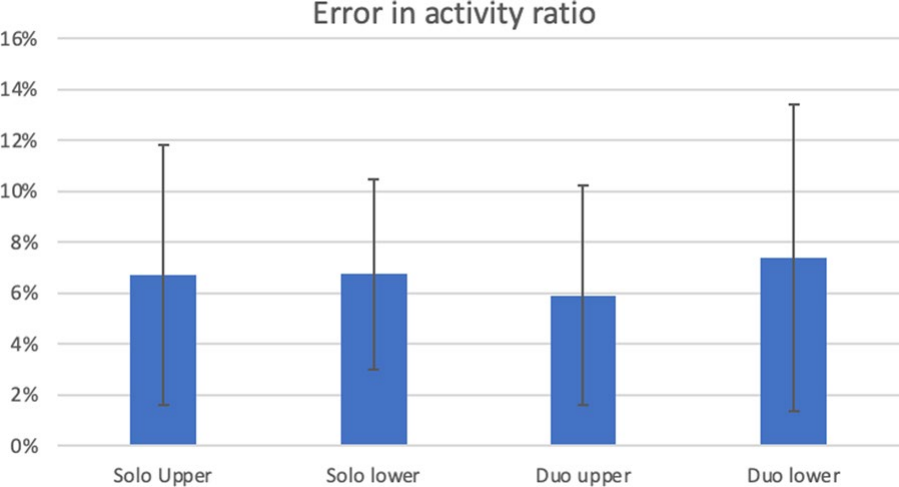
Error in activity ratio between the two phantoms according to the conditions of measurement

The activity ratio between the two fantoms measured with a well counter goes from 32 to 52% depending on the experiment. When measured on the images, the activity ratios showed an error of 6.7% ± 5.1% for Solo upper position, 6.7% ± 3.7% for Solo lower position, 5.9% ± 4.3% for Duo upper position, and 7.4% ± 6% for Duo lower position (Fig. 7).

### In vivo study

**Fig. 8:**
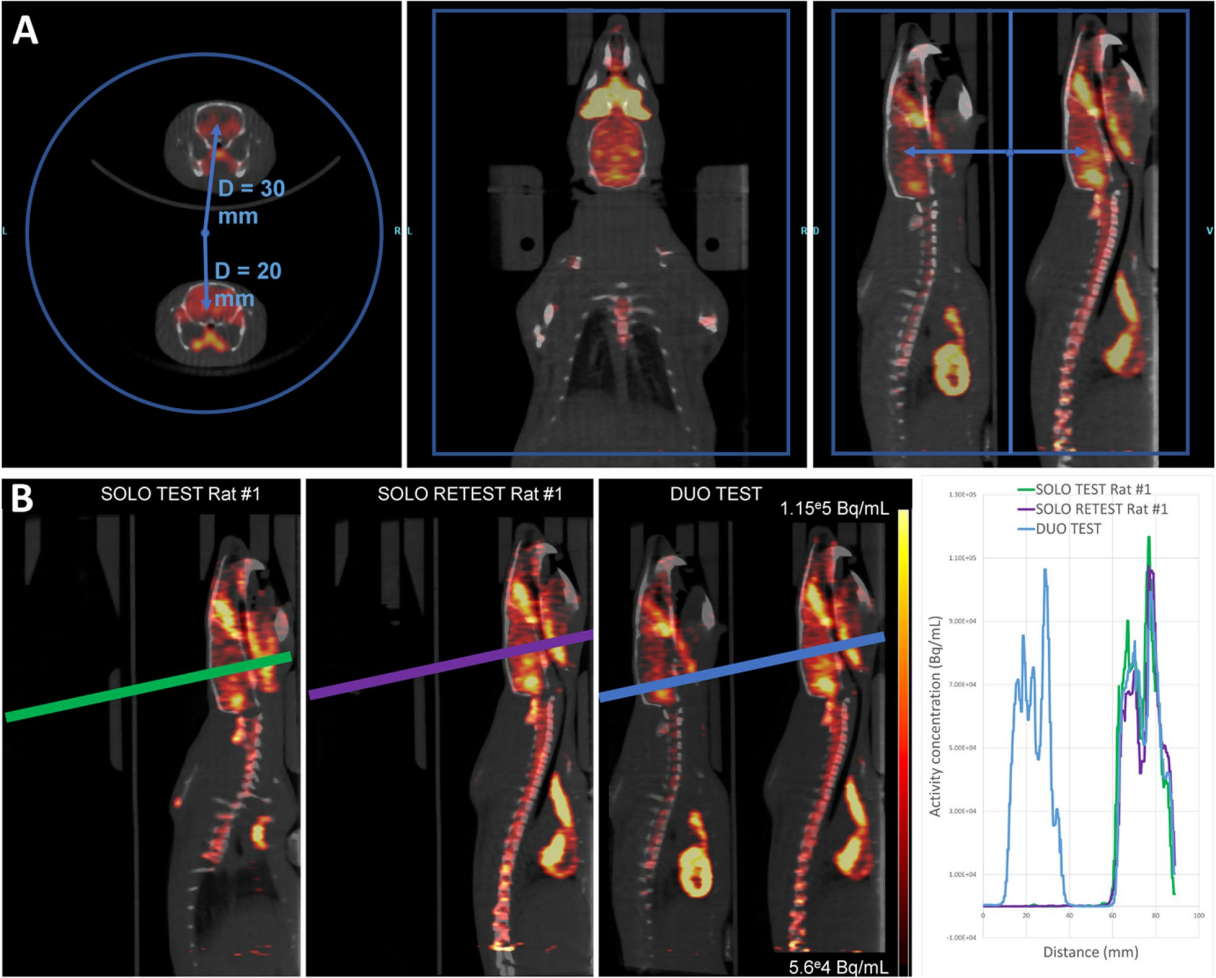
**A** Dual rat position within PET field of view. Brains at 20 and 30 mm, respectively, from the center of the FOV. **B** Sagittal slices from the reconstructed images of the same rat scanned in SOLO TEST, SOLO RETEST or DUO TEST. Color-coded profile lines were drawn through identical structures in all the scans (left) to obtain representative plots of radioactivity distribution (right)

The brain of the upper rat has an offset position of 30 mm from the center of the FOV, and the brain on the lower rat has an offset position of 20 mm (Fig. 8A), as seen in the in vitro study.

A profile line was drawn through identical structures in all scans to compare activity profiles across conditions and positioning of the rats (Fig. 8B).

**Fig. 9:**
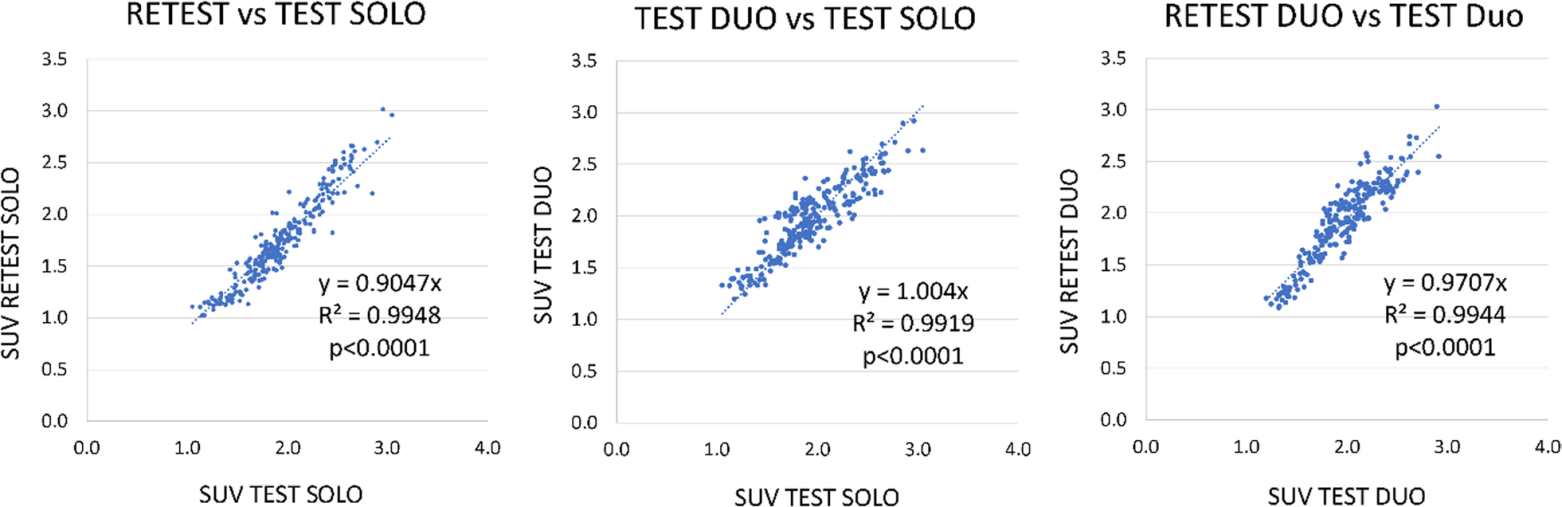
Linear regressions of quantitative PET data, comparing RETEST versus TEST SOLO, TEST DUO versus TEST SOLO and RETEST DUO versus TEST DUO

Linear regressions comparing the different configurations of scanning showed an excellent correlation with a slope near unity (from 0.90 to 0.97), a correlation factor higher than 0.99 and a *p* value lower than 0.0001 (Fig. 9).

**Fig. 10:**
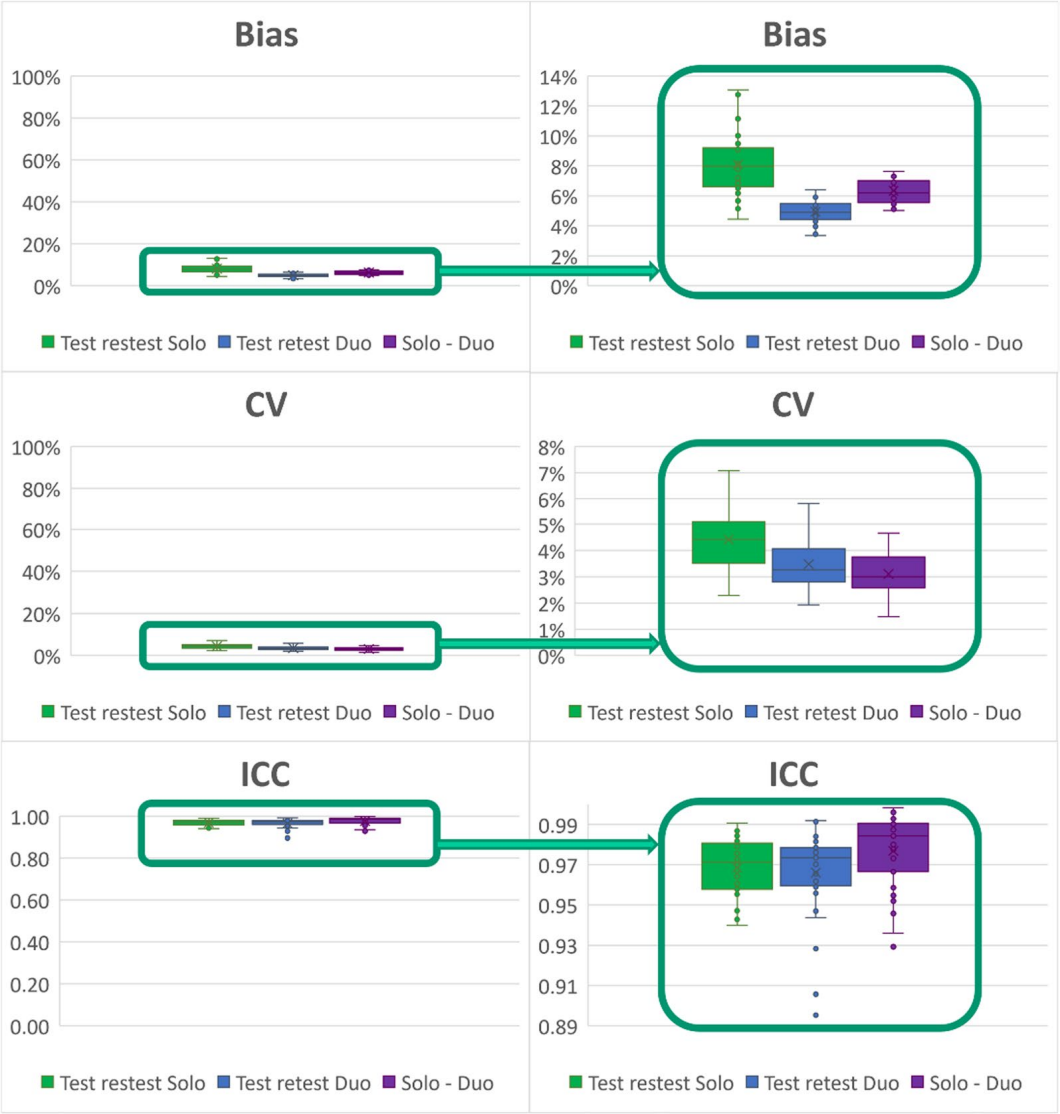
Bias, coefficient of variability (CV) and reliability assessed by the intraclass correlation coefficient (ICC). Reproducibility is assessed for test–retest with one animal in the FOV (SOLO), two animals (DUO) or one versus two animals (SOLO–DUO)

The higher reproducibility was observed in SOLO versus DUO conditions. However, good results were obtained whatever the condition with a bias between 5 and 13%, CV between 3 and 7% and ICC between 0.95 and 0.99 (Table 1 and Fig. 10).

**Table 1:**
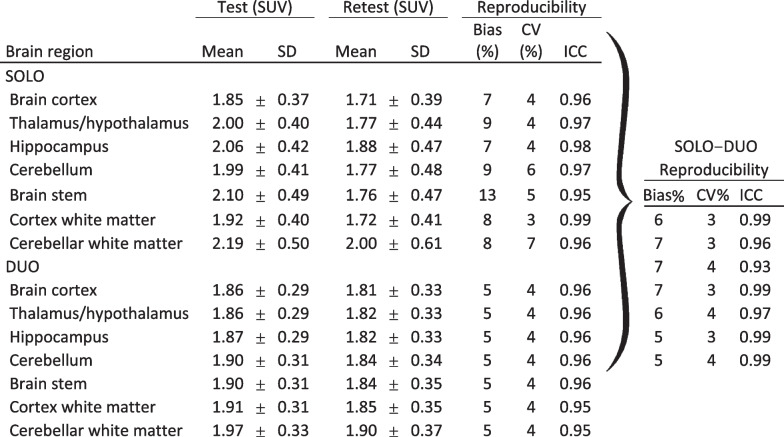
Reproducibility parameters

Reproducibility parameters in the comparisons of various conditions: Test versus retest for one animal in the same bed position (SOLO), test and retest when two animals were in the FOV (DUO), and SOLO versus DUO condition comparisons. Mean ± SD SUV in test and retest condition. Reproducibility is expressed as Bias, coefficient of variation (CV) and Intraclass correlation Coefficient (ICC)

## Discussion

We assessed the potential use of a homemade dual support system compatible with Siemens INVEON PET/CT scanner allowing the imaging of two rats simultaneously. The design of this system was based on existing systems, improving them both in terms of ergonomics and accessibility at a limited cost.

We performed scans in various configuration on phantoms and in vivo scan with rats, to evaluate a potential loss of measurement accuracy and a bias in quantitative measurement.

Scanning two rats during the acquisition reduces costs (because animals can be injected with tracer of the same production) and saves time. Because scans with dual rats do not affect the typical error of the measured parameter, it potentially divides the time by two for carrying out an experiment on a cohort size determined by power analysis.

To our knowledge, only one study [7] has previously demonstrated the feasibility of PET simultaneous multiple scanning and positional reproducibility on rat preclinical model. As the dual rat support system of Cheng et al. was designed to be used on a PET-only system (Siemens microPET P4), the animals could be positioned head-to-head since the imaging system allowed access to both sides of the FOV. The PET/CT INVEON scanner used in this work has only one side accessible for tracer injection, since the CT scanner side is shielded for radioprotection purposes. For scanning two rats simultaneously, we designed a dual support system where the two rats are positioned in the same directions on bunk beds, allowing access to both animals’ tail veins. As the positioning of each rat implies an offset from the scanner’s FOV center, one aim of our study was to evaluate its influence on the measurement accuracy.

During the experimental procedures, no behavioral difference was observed in animal conditioning. However, the only control of monitoring was the oxygen saturation level, which was not sufficient to test for a hypothetic higher concentration of gas in the lower position, due to the higher molecular weight than air. We then cannot exclude that the rat placed at the lower position might have a deeper anesthesia. This could be a limitation of our study that can be addressed in a future work.

The accuracy of the radioactivity measurements was first assessed in vitro on cylindrical phantoms (Falcon tubes) filled with 50 ml of 71.3 ± 17.5 kBq/g [^18^F]FDG solution. Experiments were performed with pair of phantoms filled with different activity: one higher and one lower (activity ratio of 32–52% between phantoms), to quantify the impact on the detection performances despite the position in the scanner (upper, lower or dual). With calibrated activity in phantoms with size equivalent to rat size, we demonstrated that the measurement accuracy is similar when performing scan with one or two emitting objects in the FOV. The 6% difference between conditions is in the range of the uncertainties of the PET measures. We also showed that dispersion of the voxel values in a ROI where activity is supposed to be homogenous is around 5% and, again, it is not increased when two objects are in the FOV. Moreover, we did not observe spillover of activity from one phantom to the other. Finally, the activity contrasts between regions are preserved even when the measurement is simultaneous.

The observations made on the accuracy of the measurements and their independence in the field of view for phantom conditions were favorable to the in vivo experimentation for which the ground truth cannot be so precisely known. In the second experiment, which was performed on living rats, the performance criterion was evaluated by the reproducibility parameters of the measurement. Indeed, the use of the bunk bed could be validated provided that the test–retest reproducibility parameters were the same with one or with two individuals in the FOV.

The results showed that the in vivo reproducibility was excellent with a bias of less than 10%, a coefficient of variation of less than 5%, and, above all, an ICC greater than 0.95, which is exceptional. This latter result means that the inter-individual variability is clearly greater than the intra-individual variability in the measurements, which guarantees that the slight variations between test and retest can be highlighted whether they are systematically reproduced between two groups or between two experimental conditions practiced on individuals from the same group. Knowing these, the test–retest reproducibility performances in SOLO scan conditions, we were able to verify that they were identical, or even better, in DUO conditions, and that it was also as good by comparing scans performed in SOLO with scans performed in DUO. In conclusion, we showed that there is no loss of quality by carrying out a scan with 2 rats simultaneously, and moreover, the data acquired with one rat in the FOV could be compared to the data acquired when two rats are in the FOV.

Although we took the precaution of performing these measurements at a relatively high radioactivity level, a limitation of our study is that we did not demonstrate whether these results are still valid in any range of radioactivity. In particular, we cannot exclude a saturation effect of the PET detector at high radioactivity. To overcome this risk, it is therefore advisable to evaluate the activity injected in each rat and to check on the performance curves of the machine that twice this activity does not reach the peak Noise Equivalent Count (NEC) that can be seen on the curve's scanner performance [10]. The nNEC peak of the INVEON camera being reached for a phantom at approximately 100 MBq, the maximum activity to be injected into an animal would be 50 MBq, which corresponds to a weight activity of 160 kBq/g for an average-sized rat 300 g. This weight activity is quite exceptional and probably never practiced. In our study, we injected a weight activity of approximately 35 kBq/g, which provided an image of quite sufficient quality.

Another risk is the saturation of the detector during dynamic acquisitions at the first pass of the tracer at a high concentration, concentrated in the arterio-venous space. This risk also exists for an acquisition with a single animal, but it is effectively doubled when two animals are in the FOV. A proposed solution is to slightly shift the injection time (~ 10 s) of the two rats so that the peaks of activity in the field of view do not overlap temporally.

Additionally, before considering using other isotopes with higher positron energy than ^18^F (^11^C, ^15^O, or ^68^Ga), a proper evaluation of the performance should be done, because of the potential spillover of the activity between the two animals.

Finally, if the technical solution we propose has been applied to a PET/CT Siemens INVEON system, it can be transposed to any other small animal system, but always with consideration of the scanner performance, specially by checking that the counting rate is compatible with the injected dose in animals.

## Conclusions

We demonstrated here the benefits of a multiple-animal PET scanning technique, allowing to minimize the total number of imaging studies required, to decrease the total scanning time, and to condense the volume of data for processing and storage and to significantly reduce the overall cost. The dual support system dedicated to simultaneous rat PET scanning confirms its utility to increase the throughput of small animal PET imaging without considerable loss of measurement accuracy and quantitative precision. In comparison with a single rat bed, cost and time associated with each scan were substantially reduced.

## Data Availability

The datasets generated during and/or analyzed during the current study are available from the corresponding author on reasonable request.

## Abbreviations

BSMSS: Mean sum of square between subjects
CT: x-ray computed tomography
CV: Coefficient of variation
FDG: FluoroDeoxyGlucose
FWHM: Full width at half maximum
ICC: Intraclass correlation coefficient
IRW: Inveon research workplace
NEC: Noise equivalent count
OP-OSEM3D: 3D ordinary poisson ordered subset expectation maximization
ROI: Region of interest
SD: Standard deviation
SpO_2_: Peripheral capillary oxygen saturation
TEP: Positron emission tomography
VOI: Volume of interest
WSMSS: Mean sum of square within subjects
